# Supplementary figures and images for: MLL4 regulates postnatal palate growth and midpalatal suture development
Source: Front Cell Dev Biol. 2025 Jan 24;13:1466948. doi: 10.3389/fcell.2025.1466948 (PMC11803150; doi:10.3389/fcell.2025.1466948)

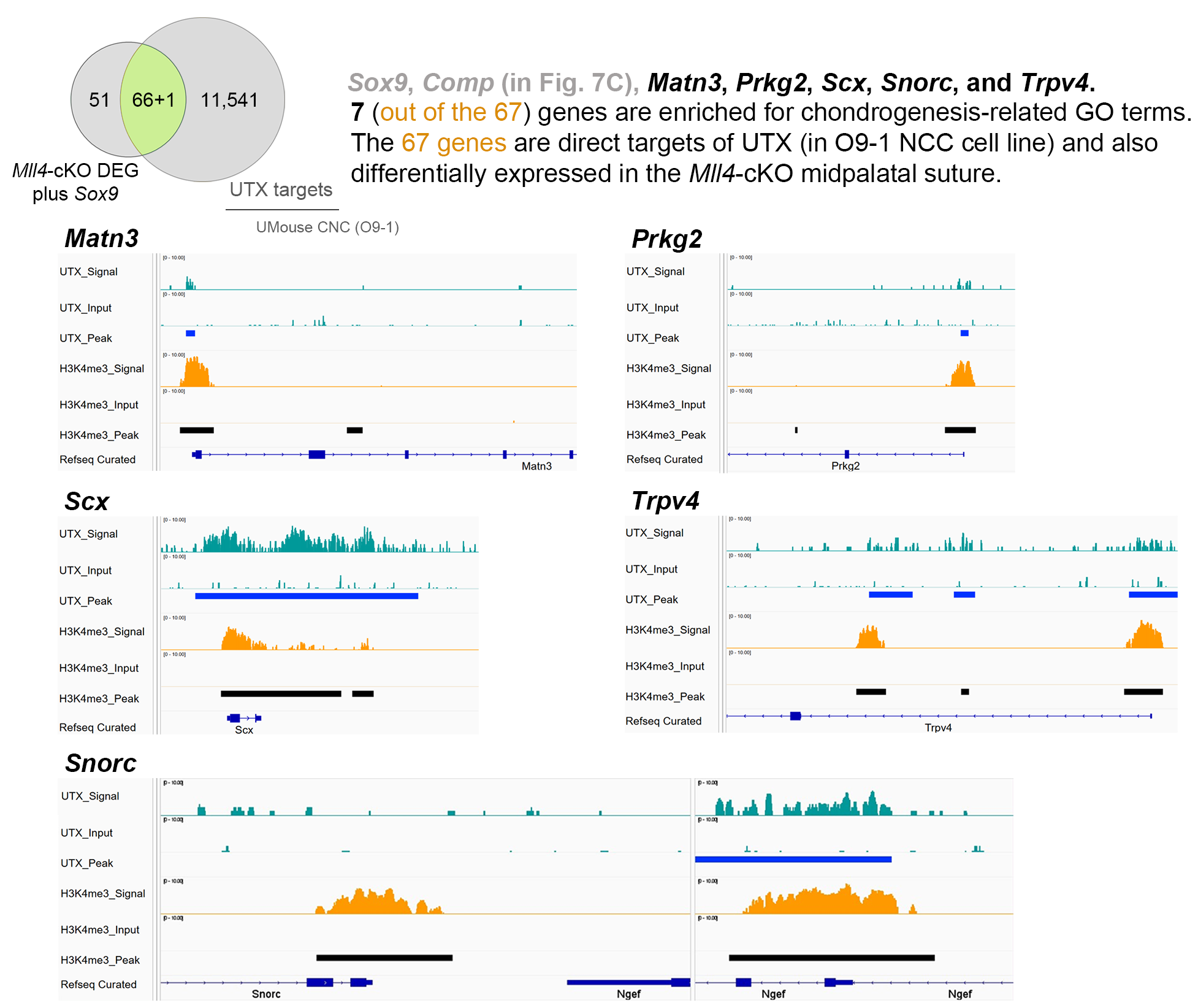

Supplement: Supplementary file 1 [file Image1.tif]
